# Construction of a prognostic risk score model based on the *ARHGAP* family to predict the survival of osteosarcoma

**DOI:** 10.1186/s12885-023-11673-w

**Published:** 2023-12-01

**Authors:** Wenda Liu, Kezhou Xia, Di Zheng, Xinghan Huang, Zhun Wei, Zicheng Wei, Weichun Guo

**Affiliations:** Department of Orthopaedics, Renmin Hospital of Wuhan University, 238 Jiefang Road, Wuhan, 430060 China Hubei Province

**Keywords:** Osteosarcoma, Prognosis model, Immune pathway infiltration, *ARHGAP* family genes, Individualized treatment

## Abstract

**Background:**

Osteosarcoma (OS) is the most common primary malignancy of bone tumors. More and more ARHGAP family genes have been confirmed are to the occurrence, development, and invasion of tumors. However, its significance in osteosarcoma remains unclear. In this study, we aimed to identify the relationship between *ARHGAP* family genes and prognosis in patients with OS.

**Methods:**

OS samples were retrieved from the TCGA and GEO databases. We then performed LASSO regression analysis and multivariate COX regression analysis to select *ARHGAP* family genes to construct a risk prognosis model. We then validated this prognostic model. We utilized ESTIMATE and CIBERSORT algorithms to calculate the stroma and immune scores of samples, as well as the proportions of tumor infiltrating immune cells (TICs). Finally, we conducted in vivo and in vitro experiments to investigate the effect of *ARHGAP28* on osteosarcoma.

**Results:**

We selected five genes to construct a risk prognosis model. Patients were divided into high- and low-risk groups and the survival time of the high-risk group was lower than that of the low-risk group. The high-risk group in the prognosis model constructed had relatively poor immune function. GSEA and ssGSEA showed that the low-risk group had abundant immune pathway infiltration. The overexpression of *ARHGAP28* can inhibit the proliferation, migration, and invasion of osteosarcoma cells and tumor growth in mice, and IHC showed that overexpression of *ARHGAP28* could inhibit the proliferation of tumor cells.

**Conclusion:**

We constructed a risk prognostic model based on five *ARHGAP* family genes, which can predict the overall survival of patients with osteosarcoma, to better assist us in clinical decision-making and individualized treatment.

**Supplementary Information:**

The online version contains supplementary material available at 10.1186/s12885-023-11673-w.

## Introduction

Osteosarcoma (OS) is one of the most common malignancies in the skeletal system. It originates in the mesenchymal tissue and is most likely to occur in the long diaphyseal region with abundant blood supply, such as the distal femur and proximal tibia [1]. Most osteosarcomas are primary and a few are secondary [2]. Osteosarcoma mainly occurs in children and adolescents with strong bone growth and development. Due to the highly invasive nature of osteosarcoma, 75% of patients with osteosarcoma have distant metastases, mainly to the lung, and the prognosis is poor, with an overall survival rate of less than 20% [3–5]. At present, the basic treatment techniques for osteosarcoma are neo-adjuvant chemotherapy and surgical resection, which can enhance the survival percentage of patients with an early diagnosis of osteosarcoma by 60–70% [6]. With the development of molecular biology and tissue bioengineering, great progress has been made in treating osteosarcoma, which has significantly improved the postoperative quality of life and the 5-year survival rate of patients with malignant tumors [2]. However, those with metastatic tumors who cannot be treated with surgery have a very poor prognosis, with a 5-year survival rate of less than 20% [7]. Due to the high heterogeneity and low incidence of osteosarcoma, it is difficult to identify a specific driver gene, so we currently cannot predict the prognosis of patients based on the changes of a single molecule in their body [8]. Therefore, there is an urgent need for some biomarkers to predict the survival status of patients with clinical osteosarcoma to better assess the risk and personalized management of patients.

*Rho* family is a member of the Ras supergene family of guanosine triphosphatase (*GTPase*), which is involved in cell morphology, gene transcription, cell cycle, cell apoptosis, cell carcinogenesis, cell migration and infiltration, and other processes [9, 10]. However, the *Rho* GTPase-activating proteins (*RHOGAPs*) family is a negative regulatory factor of the *Rho* family proteins [11]. We have found *ARHGAP* proteins to be altered in expression in many diseases, including cancer, so they may be a potential target for treating diseases [12, 13]. They have reported that the high expression of *ARHGAP* protein can inhibit the proliferation and migration of tumor cells, and has a good prognosis for mice, so it may become a target for tumor therapy [14]. However, there have been no systematic studies on whether *ARHGAP* protein affects the prognosis of patients with osteosarcoma and its clinical significance.

In this study, we have studied the *ARHGAP* family genes systematically. We identified the genes associated with the prognosis of osteosarcoma in *ARHGAP* and selected five genes to construct a unique prognostic risk model for predicting the survival time of patients with osteosarcoma. Then we validated the prognostic results in the training cohort, testing cohort, entire cohort, and GSE39055 cohort, respectively. Finally, we studied the relationship between our constructed prognostic model and the immune microenvironment. Our study is more helpful in evaluating the prognosis of patients with osteosarcoma and conducting individualized treatment.

## Materials and methods

### Data acquisition

We collected RNA sequencing data (RNA-seq) in fragment per kilobase method (FPKM) format and matching clinical information from The Cancer Genome Atlas (https://portal.gdc.cancer.gov/) for 88 patients with osteosarcoma. Clinical data of these patients should include age, sex, survival status, follow-up time, diagnosis time, etc. To make the results more reliable, we downloaded the GSE39055 dataset from the Gene Expression Omnibus database (https://www.ncbi.nlm.nih.gov/geo/) for external validation. This dataset contains patient RNA-seq data and corresponding clinical information. For the convenience of follow-up analysis, we excluded those patients with no follow-up information or unknown survival status and finally included a total of 123 patients for the follow-up study, including 86 patients in the TARGET database and 37 patients in the GSE39055 dataset. Then, we used the “sva” package in R to process the two data sets to eliminate the batch effect.

### Identification and differential expression analysis of prognostic-related ARHGAP family genes

We got the *ARHGAP* family gene from the GeneCards database (https://www.genecards.org/) and previous research [11]. We then performed a univariate Cox regression analysis based on the expression of these genes and patient clinical information, and those genes with *p* < 0.05 were considered to be prognostic-related. According to the expression amount of these genes, we drew the expression heatmap of each gene in each sample and the expression correlation between them.

### Construction of prognostic risk profiles based on the ARHGAP family and subsequent validation

To construct a prognostic risk model and for further verification, we randomly divided patients in the TARGET cohort 1: 1 into a training cohort and a testing cohort. We then performed multivariate Cox regression and the Least Absolute Shrinkage and Selection Operator (LASSO) regression analysis for prognostic-related genes in the *ARHGAP* family in the training cohort. Based on this result, we identified the *ARHGAP* family genes and their corresponding regression coefficients that ultimately participated in the construction of the prognosis model. We used the following formula to calculate the risk score for each osteosarcoma patient in the cohort: Risk score = ${\sum }_{k=1}^{n}( Coefficient\left(i\right)*Expr(i))$, where Coefficient is the regression coefficient, Expr is the expression of *ARHGAPs* and n is the number of genes we included in the prognostic model. Using the same formula, we can determine the respective risk coefficients for the testing cohort, the entire internal cohort, and the external GSE39055 cohort. We divided osteosarcoma patients in each cohort into high—and low-risk groups based on their risk scores. We did a survival analysis of the training cohort, the testing cohort, the entire internal cohort, and the external GSE39055 cohort to assess survival differences between high- and low-risk groups in each cohort. To confirm the sensitivity, specificity, and accuracy of the risk-prognosis model, we simulated the time-dependent receiver operating characteristic (ROC) curve.

### Construct a nomogram to verify and predict the prognosis of osteosarcoma patients

A nomogram was created by combining risk score and other two clinicopathological characteristics including gender, and age in the TCGA cohort. We were then using the nomogram to predict 2-year, 3-year, and 5-year survival for osteosarcoma. Time-dependent ROC curves and calibration curves were simulated in the TCGA and GSE39055 datasets, respectively, to verify the efficacy of the nomogram in predicting the overall survival of patients with osteosarcoma.

### Screening of differential genes between high- and low-risk groups

We have divided patients in the TCGA entire cohort into high- and low-risk groups. We secured the differentially expressed genes (DEGs) between the two groups, and the screening standard was | log2FC |> 0.5 and *p*-value < 0.05. Then we made a heatmap based on the expression of different differential genes in each sample. We generated the hub genes by simulating the PPI network of differential genes between the two groups using The Search Tool for the Retrieval of Interacting Genes/Proteins (STRING) web-based database (string-interaction.org).

### Functional enrichment analysis between differentially expressed genes

Gene enrichment analysis can help us identify which biological functions and pathways are primarily responsible for prognostic risk scores between the two groups. Next, we carried out the Kyoto Encyclopedia of Genes and Genomes (KEGG) pathway analysis and Gene Ontology (GO) enrichment analyses to analyze these differential genes [15–17]. To have a more intuitive and in-depth understanding of the mechanism of differential genes, we then conducted a GSEA enrichment analysis on the differential genes between the high- and low-risk groups to determine whether they play a role in a biological process or pathway. We analyzed the standard genetic set "c2.cp.kegg.v7.0.symbols.gmt" by using GSEA software.

### Analysis of immune function between high- and low-risk group

To understand whether the prognostic risk score, we constructed works in the tumor microenvironment (TME), we calculated the immunological scores, estimate scores, stromal scores, and tumor purity in the immune microenvironment of osteosarcoma patients between the two risk groups. We used the CIBERSORT algorithm to analyze the expression data of each sample and calculate the relative abundance of 22 types of immune cells in them [18, 19], and then used R packets to visualize the abundance of immune cells between the two risk groups. We also analyzed the correlation between each immune infiltrating cell in the osteosarcoma sample. Then we performed single-sample gene set enrichment analysis (ssGSEA) in the TCGA and GSE39055 datasets respectively and obtained the differences in immune function scores and expression of 22 kinds of infiltrated cells between high- and low-risk groups.

### Cell line culture and transfection

We obtained the 143B and U2OS human osteosarcoma cell lines from the China Center for Type Culture Collection (Wuhan, China). To culture these cell lines, we used RPMI-1640 medium (Invitrogen, USA) supplemented with 10% fetal bovine serum (FBS; Tian Hang, China) and 1% Penicillin–Streptomycin and incubated them at 37 °C with 5% CO2. The *ARHGAP28* expression plasmids were purchased from Sangon Biotech Co., Ltd (Shanghai, China), and we cultured OS cells in 6-well and 96-well plates for subsequent experiments. We conducted a series of assays, including western blot analysis, CCK-8 assay, transwell invasion assay, and wound healing assay, using *ARHGAP28*-overexpressing cells.

### Western blot analysis

We extracted total proteins from osteosarcoma cells in good growth condition using RIPA buffer (Servicebio Technology, Wuhan, China) and followed the instructions. The protein concentration was quantified using a BCA kit (Servicebio Technology, Wuhan, China). Next, the total protein was separated by electrophoresis and transferred to a membrane. After blocking and washing the membrane three times with tris-buffered saline with tween (TBST), we cut the membrane and incubated primary antibodies overnight at 4 ℃. Following that, the membrane was washed and incubated with secondary antibodies. Finally, we used the ECL kit (Thermo Fisher Scientific) for display.

### CCK-8 assay

We inoculated osteosarcoma cells in good growth condition on 96-well plates, with three multiple wells in each group. We replaced the complete medium, which contained 10 μL CCK-8 reagent, at 0, 24, 48, 72, 96, and 120 h after planting the plates and incubated them for 2 h each time. We then used a microplate reader (Bio-Rad Laboratories, Inc.) to measure the absorbance of each well at OD 450 nm (optical density), which indicates the viability of each cell line.

### Transwell invasion assay

Transwell chambers (Corning, USA) and Matrigel (Corning, USA) were used for conducting invasion experiments. The upper chamber was filled with the medium at a 1:6 ratio. After coagulation, 200 µL of medium containing 1 × 10^5^ cells was added to the upper layer of each chamber, and 600µL of complete medium (containing 10%FBS) was added to the lower layer of each chamber. After 48 h of cell culture, the cells were removed and fixed with 4% paraformaldehyde and stained with 1% crystal violet. Take pictures under an inverted microscope (Olympus, Japan) and count the number of cells.

### Wound healing assay

We inoculated osteosarcoma cell lines 143B and U2OS into 6-well plates with 2 mL complete medium added to each well and 3 multiple Wells in each group. When cell density reached 95%, a 100µL gun tip was used to mark the bottom of each well of the culture plate, and serum-free medium was replaced, and the culture was continued after photographing with an inverted microscope (Olympus, Japan). After 24 and 48 h of culture, the area of intermediate scratches was observed with an inverted microscope and photographed. The wound area was measured using the ImageJ software and wound healing percentage was calculated to evaluate the migrate ability. The calculation formula is below: Migration rate = (area of 0 h—area of 24 h or 48 h) / (area of 0 h) × 100.

### Animal studies

The Renmin Hospital of Wuhan University Ethics Committee authorized the experiments. Female BALB/c nude mice (4–6 weeks old) were obtained from Beijing HFK Experiment Animal Center (Beijing, China) and randomly divided into two groups of six mice each (NC, *ARHGAP28* OE). These mice were subcutaneously injected with stably transfected 143B osteosarcoma cells and kept in a standard environment with food and water. Tumor size and volume were monitored weekly. After four weeks, all mice were euthanized with 2% pentobarbital sodium (150 mg/kg), and tumors were excised and weighed. The tumors were then either preserved in liquid nitrogen or fixed in 4% paraformaldehyde. The care of the laboratory animals and animal experiments were performed following the animal ethics guidelines and approved protocols of Renmin Hospital of Wuhan University.

### Immunohistochemistry (IHC) staining

The tumor tissue was fixed with 4% paraformaldehyde for 24 h and cut into 4-µm slices, which were then blocked with 1% bovine serum albumin at room temperature for 1 h. After that, the slices were incubated with corresponding primary antibodies at 4 ℃ overnight, followed by 1-h incubation with secondary antibodies at room temperature. Finally, chromogenic detection was performed using a DAB kit (CST, USA) and observed under an inverted microscope (Olympus).

### Statistical analysis

All our data processing and picture drawing was carried out by using R software (version 4.2.1). We used the log-rank test for the Kaplan–Meier survival difference analysis of the high—and low-risk groups. We used the Wilcoxon rank-sum test and the two-tailed Student’s t-test to compare the high- and low-risk groups. Multivariate Cox regression analysis was used to identify factors that could independently predict the prognosis of patients with osteosarcoma. We defined *p* < 0.05 as a significant difference. “*” is equal to “*p* < 0.05”, “**” is equal to “*p* < 0.01” and “***” is equal to “*p* < 0.001”.

## Results

### Characterization of ARHGAP family genes

**Fig. 1:**
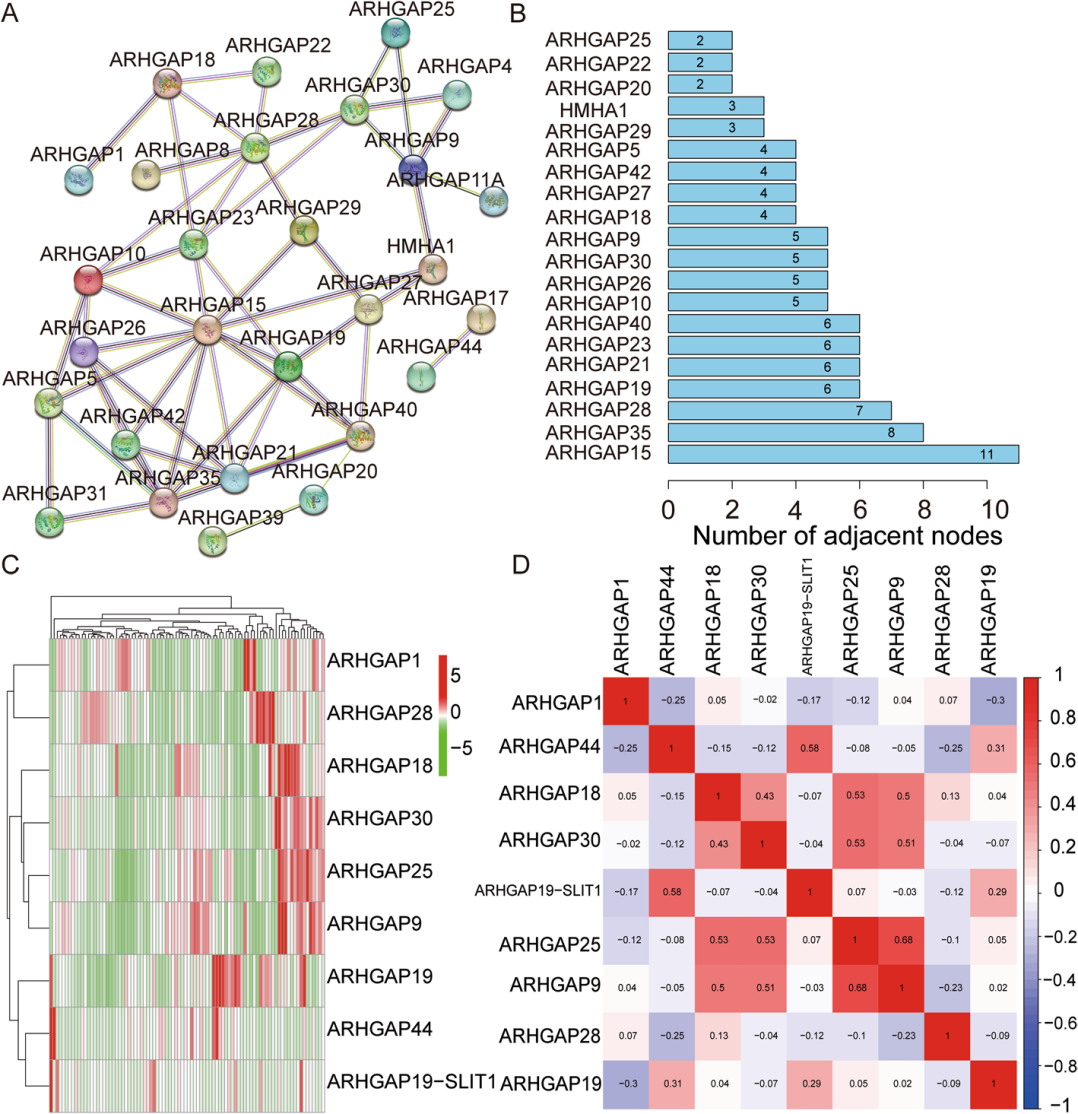
Identification of prognosis-related *ARHGAP* family genes in osteosarcoma. **A** A network of protein–protein interactions involving all *ARHGAP* genes. **B** The PPI network's hub genes. **C** Heatmap illustrating the expression patterns of prognosis-related *ARHGAP* family genes in TCGA datasets. **D** Heatmap of the genes in the *ARHGAP* family that are associated with prognosis

We included 35 genes in the *ARHGAP* family. We analyzed the expression patterns of these genes using the STRING database (Fig. 1A). Hub gene analysis suggested that *ARHGAP15*, *ARHGAP35*, *ARHGAP28*, *ARHGAP19*, *ARHGAP21*, *ARHGAP23*, *ARHGAP40*, *ARHGAP10*, *ARHGAP26*, *ARHGAP30,* and *ARHGAP9* were identified as hub genes, with the largest interaction network among these proteins (Fig. 1B). We used univariate Cox regression analysis on datasets from the TCGA database to identify prognosis-related genes of the *ARHGAP* family in osteosarcoma. Nine *ARHGAP* genes with p-values less than 0.05, as determined by univariate Cox regression analysis, were identified as *ARHGAP* family prognosis-related genes. The expression profiles of *ARHGAP* family prognosis-related genes (Fig. 1C). We show an association between prognostic genes in the *ARHGAP* family (Fig. 1D).

### Construction of a prognostic risk score model

**Fig. 2:**
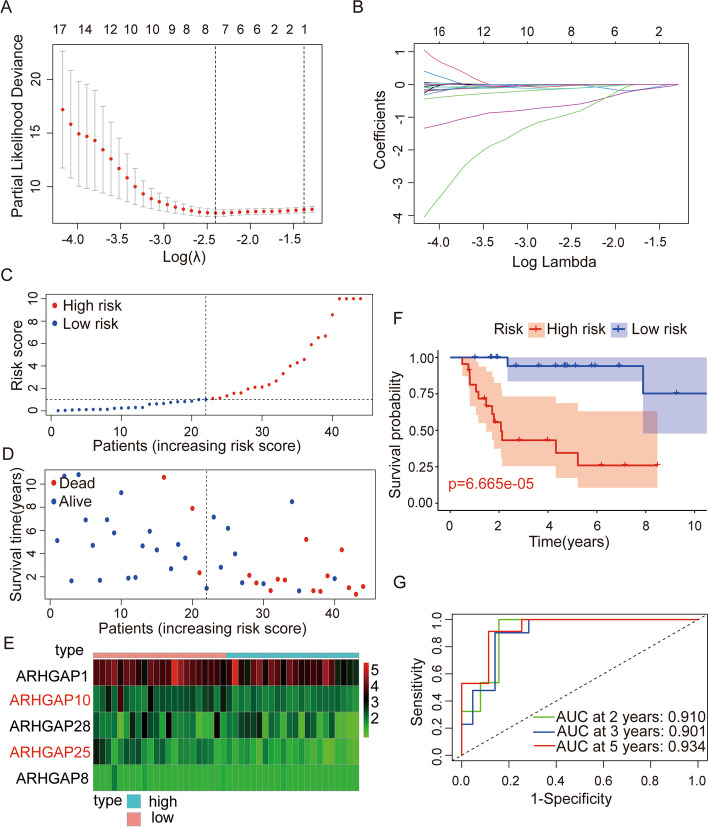
Development of prognostic risk assessment model. **A**, **B** Multivariate Cox analysis with LASSO regression. **C**, **D** Risk scores and distribution of OS patients in the training cohort. **E** Heatmap of the prognostic model genes in the training cohorts. **F** Comparison of overall survival between the two risk groups in the training cohort. The results showed a significantly different overall survival in the high- and low-OS groups. **G** Time-dependent ROC curve analysis in the training cohort

To construct a prognostic signature based on *ARHGAP* family genes, we randomly classified the TCGA OS cohort into training (*n* = 45) and testing (*n* = 43) cohorts. In the training cohort, we performed multivariate Cox and LASSO analysis to screen *ARHGAP* family genes (Fig. 2A–B). The ordinate in the Fig. 2A is Binomial Deviance. There are two dashed lines in Fig. 2A, the left is the line with the lowest error, and the right is the line with few features. Each curve in Fig. 2B represents the change trajectory of each independent variable coefficient, the upper abscissa is the number of non-zero coefficients in the model at this time. As the λ value changes, the later the coefficient is compressed to 0, the more important the variable. Five *ARHGAP* genes (*ARHGAP1*, *ARHGAP10*, *ARHGAP25*, *ARHGAP28,* and *ARHGAP8*) with the best lambda value were retained following LASSO analysis to construct a prognostic signature for OS. The risk score based on the prognosis signature was obtained using a linear combination of the expression levels of the selected genes and their corresponding coefficients. The formula was as follows: Risk score = *ARHGAP1* × (-0.05243) + *ARHGAP10* × (-0.3089) + *ARHGAP25* × (-1.09414) + *ARHGAP28* × (-2.17281) + *ARHGAP8* × (-0.26809). We then calculated the risk score for each patient in the training cohort, using the median risk score to classify patients into high- and low-risk categories. The prognosis of OS specimens declined as risk scores rose, as seen by the risk score plot (Fig. 2C–D). The heatmap shows the expression of *ARHGAP1*, *ARHGAP10*, *ARHGAP25*, *ARHGAP28*, and *ARHGAP8* in high- and low-risk groups (Fig. 2E). The Kaplan–Meier survival analysis done to evaluate overall survival between the two patient groups revealed that patients in the high-risk group had a significantly worse prognosis than those in the low-risk group (Fig. 2F). In addition, the ROC curve of the training cohort showed reliable results, with a 2-year Area Under Curve (AUC) of 0.910, 3-year AUC of 0.901, and 5-year AUC of 0.934 (Fig. 2G), indicating that *ARHGAP*-based prognostic features have good accuracy and specificity in predicting overall survival of osteosarcoma patients.

### Validation of a five-gene prognostic model from the ARHGAP gene family in an internal cohort

**Fig. 3:**
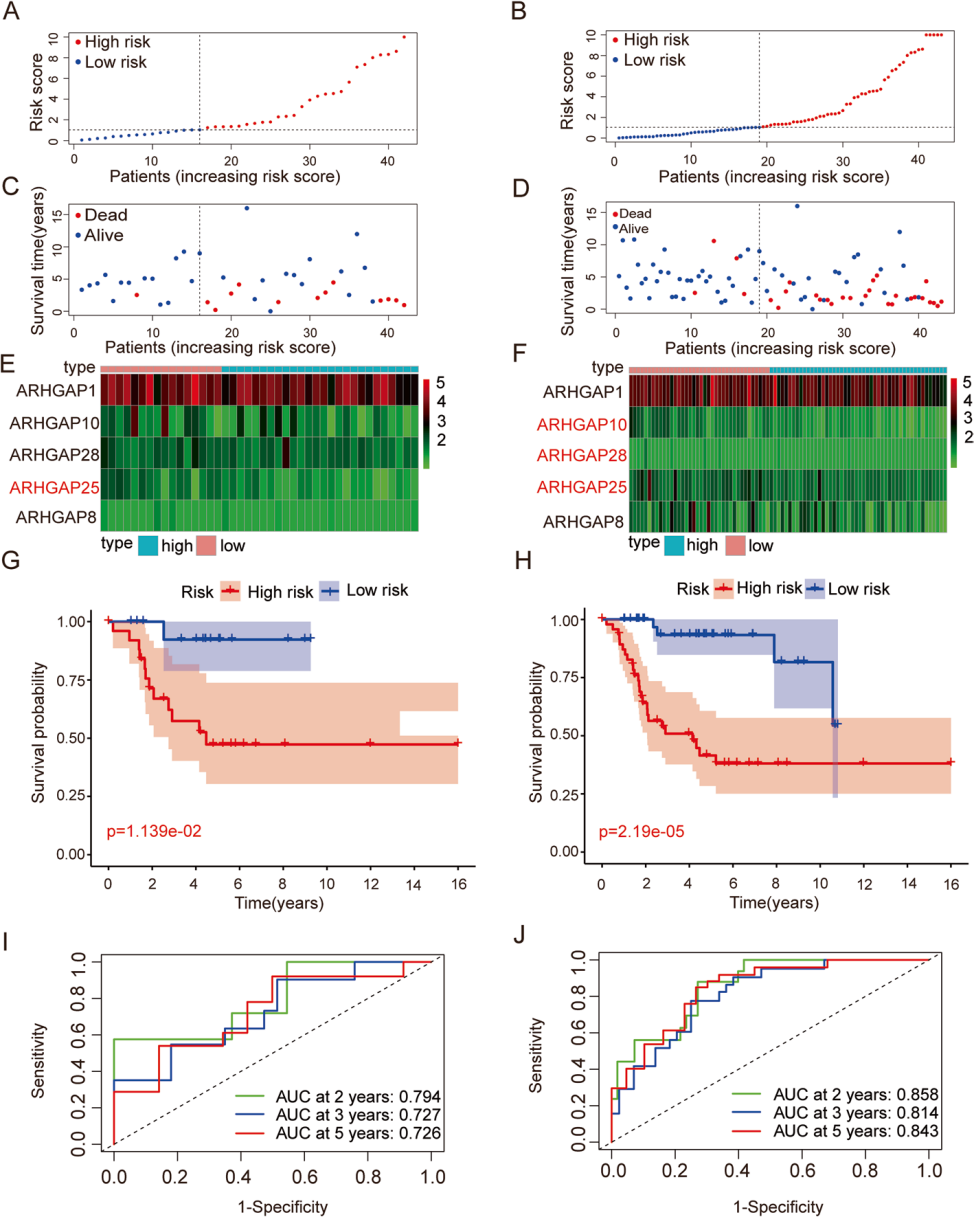
Validation of the *ARHGAP* gene signature in the testing cohort and the entire cohort. **A**, **B** The distribution of risk scores in the testing cohort and the entire cohort. **C**, **D** The distribution of survival time and status in the testing cohort and the entire cohort. **E**, **F** Heatmap of prognostic model genes in the testing cohort and entire cohort. **G**, **H** Comparison of the overall survival in the testing cohort and the entire cohort between the two risk groups. The results show a significantly different overall survival in high- and low-OS groups. **I**, **J** Time-dependent ROC curve analysis in the testing cohort and the entire cohort

We validated prognostic risk profiles based on *ARHGAP* in the testing cohort and the entire cohort to test the accuracy and repeatability of signatures. The above algorithm was used to determine the risk score of the patients, and the median risk score of the training cohort was used to divide the patients into high- and low-risk groups. We showed the distribution of the risk scores for the testing cohort and the entire cohort in Fig. 3A-B. Patients with higher risk scores had poorer outcomes (Fig. 3C-D), suggesting that the prognostic risk model we constructed can accurately predict overall survival in patients with OS. Based on the expression of five genes (*ARHGAP1*, *ARHGAP10*, *ARHGAP25*, *ARHGAP28*, *ARHGAP8*) in the above *ARHGAP* family in the testing cohort and the entire cohort, we constructed a heatmap (Fig. 3E-F). The expressions of *ARHGAP10*, *ARHGAP25,* and *ARHGAP28* were considerably greater in the low-risk group than in the high-risk group (*p* < 0.05) (Fig. S1). The Kaplan–Meier survival analysis showed a significant difference in overall survival between the two risk groups (Fig. 3G-H), and the high-risk group had a shorter survival, which further verified that the model was constructed could well predict the survival of patients. In addition, the ROC curve of the testing cohort and the entire cohort showed the same results, with a 2-year AUC of 0.794, 3-year AUC of 0.727, and 5-year AUC of 0.726 in the testing cohort (Fig. 3I), and 2-year AUC of 0.858, 3-year AUC of 0.814 and 5-year AUC of 0.843 in the testing cohort (Fig. 3J). This is consistent with our findings in the training cohort.

### Validation of a five-gene prognostic model from the ARHGAP gene family in an external cohort

**Fig. 4:**
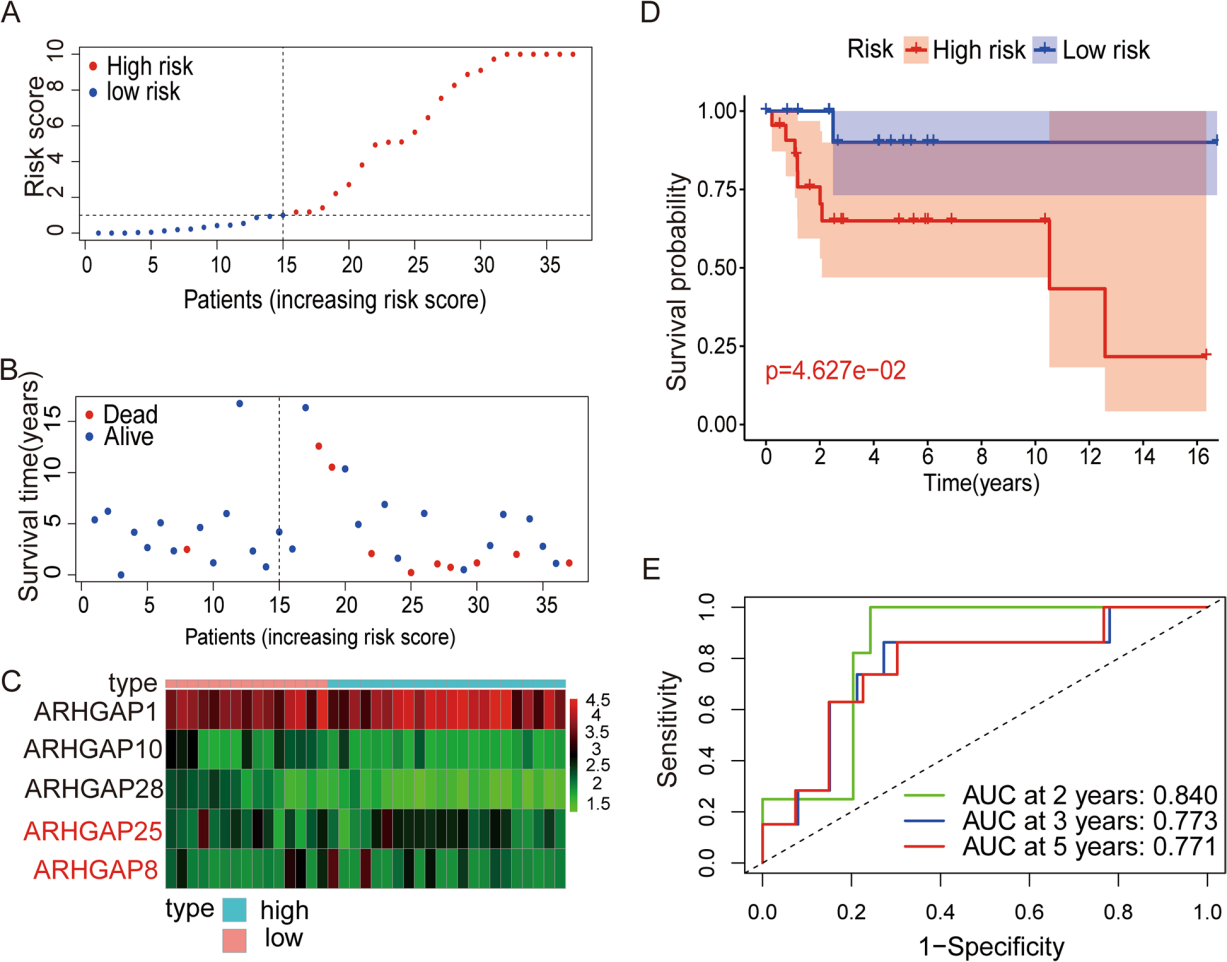
Validation of the *ARHGAP* gene signature in the GSE39055 cohort. **A** The profile of risk score in the GSE39055 cohort. **B** The distribution of survival time and status in the GSE39055 cohort. **C** Heatmap of prognostic model genes in GSE39055 cohort. **D** Comparison of the overall survival in the GSE39055 cohort between the two risk groups. The results show a significantly different overall survival in high- and low-OS groups. **E** Time-dependent ROC curve analysis in the GSE39055 cohort

We repeated this process with the GSE39055 (*n* = 37) dataset for external validation to test the accuracy and repeatability of the signature based on the five genes in the *ARHGAP* family. Following the previous steps, we divided the patients in GSE39055 into high- and low-risk groups (Fig. 4A). Similarly, we can intuitively see the distribution of survival time and survival status of patients in the GSE39055 dataset (Fig. 4B). The results showed that high-risk patients had a significantly higher mortality rate than low-risk patients. We mapped the expression heatmap of the five genes of the *ARHGAP* family used to construct the prognostic risk score in the GSE39055 dataset (Fig. 4C). Patients in the high-risk group had a lower rate of survival than those in the low-risk group, according to the Kaplan–Meier analysis (Fig. 4D), confirming the accuracy and universality of our model construction. By calculating the AUC of the GSE39055 dataset, we could further evaluate the prediction accuracy of the prognosis model we constructed, with a 2-year AUC of 0.840, 3-year AUC of 0.773, and 5-year AUC of 0.771 in the GSE39055 dataset (Fig. 4E). The results of the analysis in the external cohort are consistent with the above results, indicating that a risk-prognosis model based on the *ARHGAP* family is accurate in predicting the survival time and survival outcome of patients with osteosarcoma.

### Validate the risk prognosis model based on clinical information

**Fig. 5:**
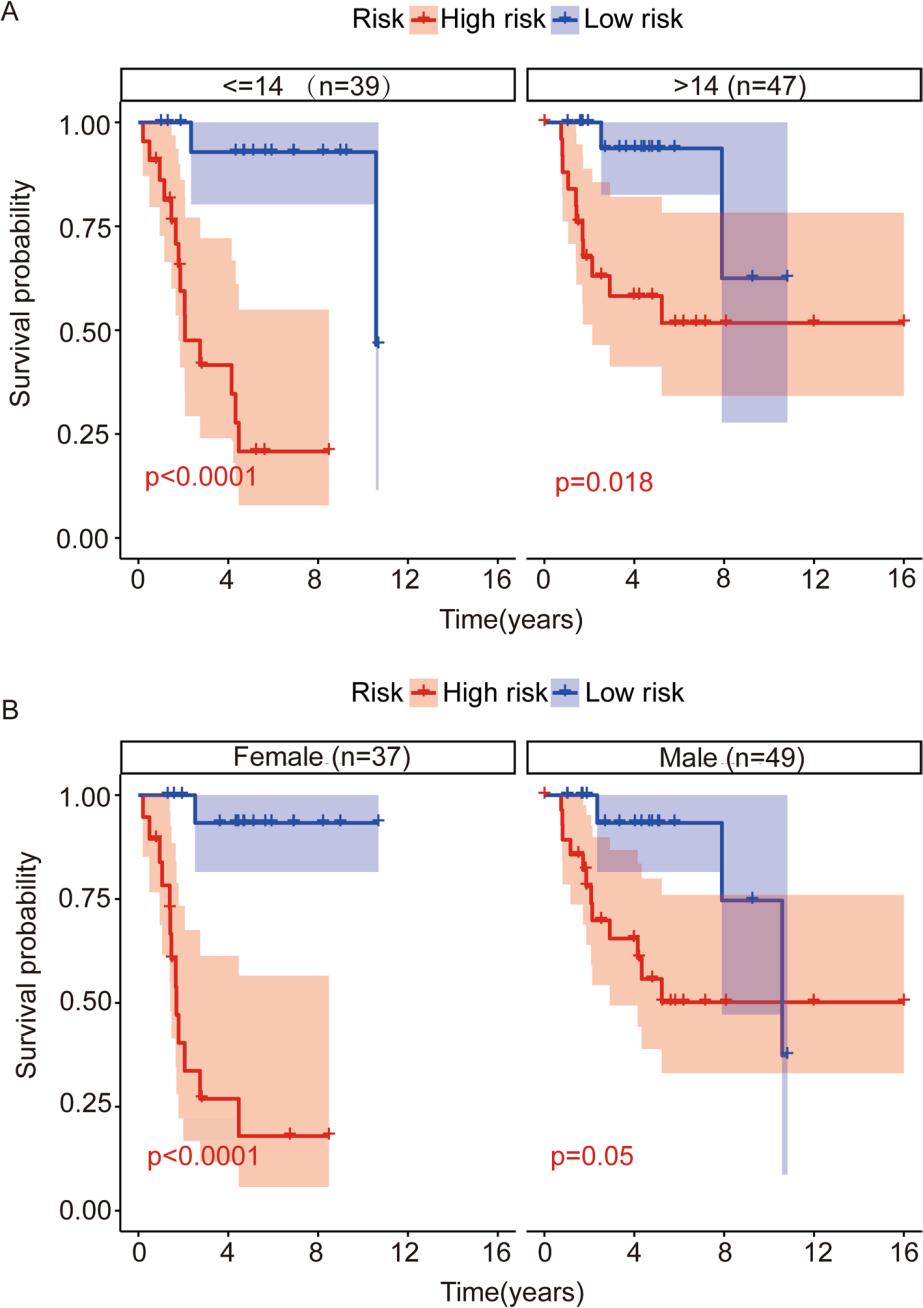
Kaplan–Meier survival curves in subgroup analyses based on various clinical variables in TCGA and GSE39055 cohort. **A** Subgroup survival analysis of risk model per age in TCGA cohort. **B** Subgroup survival analysis of risk model per gender in TCGA cohort. **C** Subgroup survival analysis of risk model per age in GSE39055 cohort. **D** Subgroup survival analysis of risk model per gender in GSE39055 cohort

We grouped patients in the TCGA entire cohort according to clinical traits such as gender (male (*n* = 49), female (*n* = 37)) and age (< = 14 (*n* = 39), > 14 (*n* = 47)), and then whether risk scores were equally applicable when discussed to different subgroups. We compared the overall survival of patients in each subgroup of the two data sets (Fig. 5A-B), and the results showed that in each subgroup, the survival of patients in the low-risk group was always longer than that in the high-risk group, and in the TCGA dataset, the difference in the risk score among the subgroups was significant (*P* < 0.05).

### Construction and validation of a Nomogram of a risk prognosis model based on the ARHGAP family

**Fig. 6:**
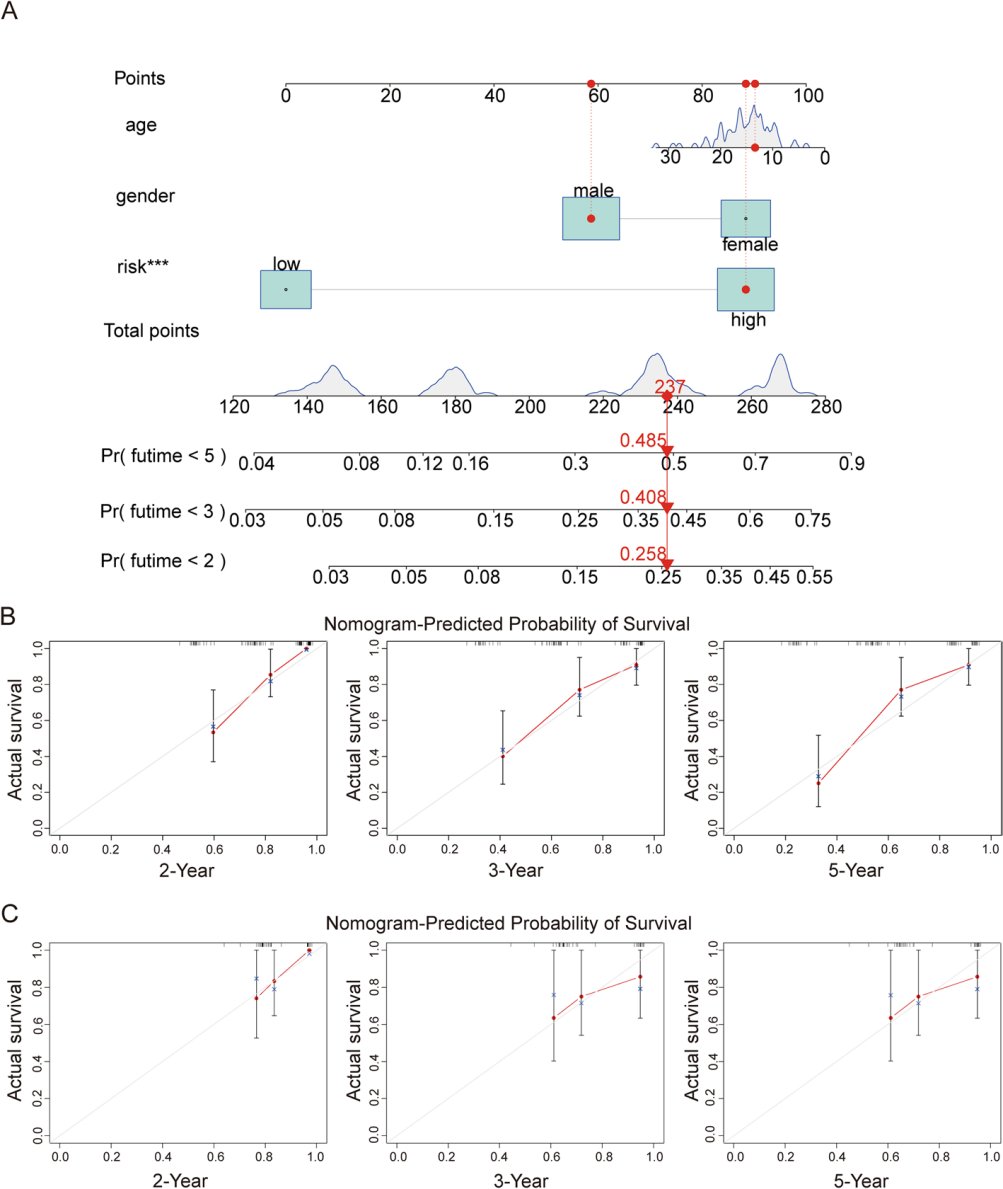
A nomogram was conducted to predict the overall survival of OS patients in the GSE39055 and TCGA cohorts. **A** Nomogram for estimating overall survival in OS patients. **B**, **C** The calibration curves for 2-, 3-, and 5-year overall survival prediction in TCGA entire cohort and external GSE39055 cohort. The nomogram-predicted survival curves were close to the 45-degree diagonal, showing that our nomogram can accurately predict the prognosis of clinical osteosarcoma patients

To make our Nomogram predictions more accurate, we incorporated clinical factors such as patient gender and age into the prognostic features used to construct them (Fig. 6A). To visualize the function of the Nomogram, we simulated a calibration curve for the 2-year, 3-year, and 5-year overall survival of patients in the TCGA entire cohort (Fig. 6B) and external GSE39055 cohort (Fig. 6C). In this figure, the 45° diagonal represents the best prediction ability. The more our calibration curve fits, the better the prediction function of our Nomogram is constructed. Calibration curves in the TCGA entire cohort and external GSE39055 cohort showed that we constructed the Nomogram could accurately assess the risk and prognosis of patients with clinical osteosarcoma.

### DEGs, PPI network, GO, and KEGG enrichment analyses in two risk groups

**Fig. 7:**
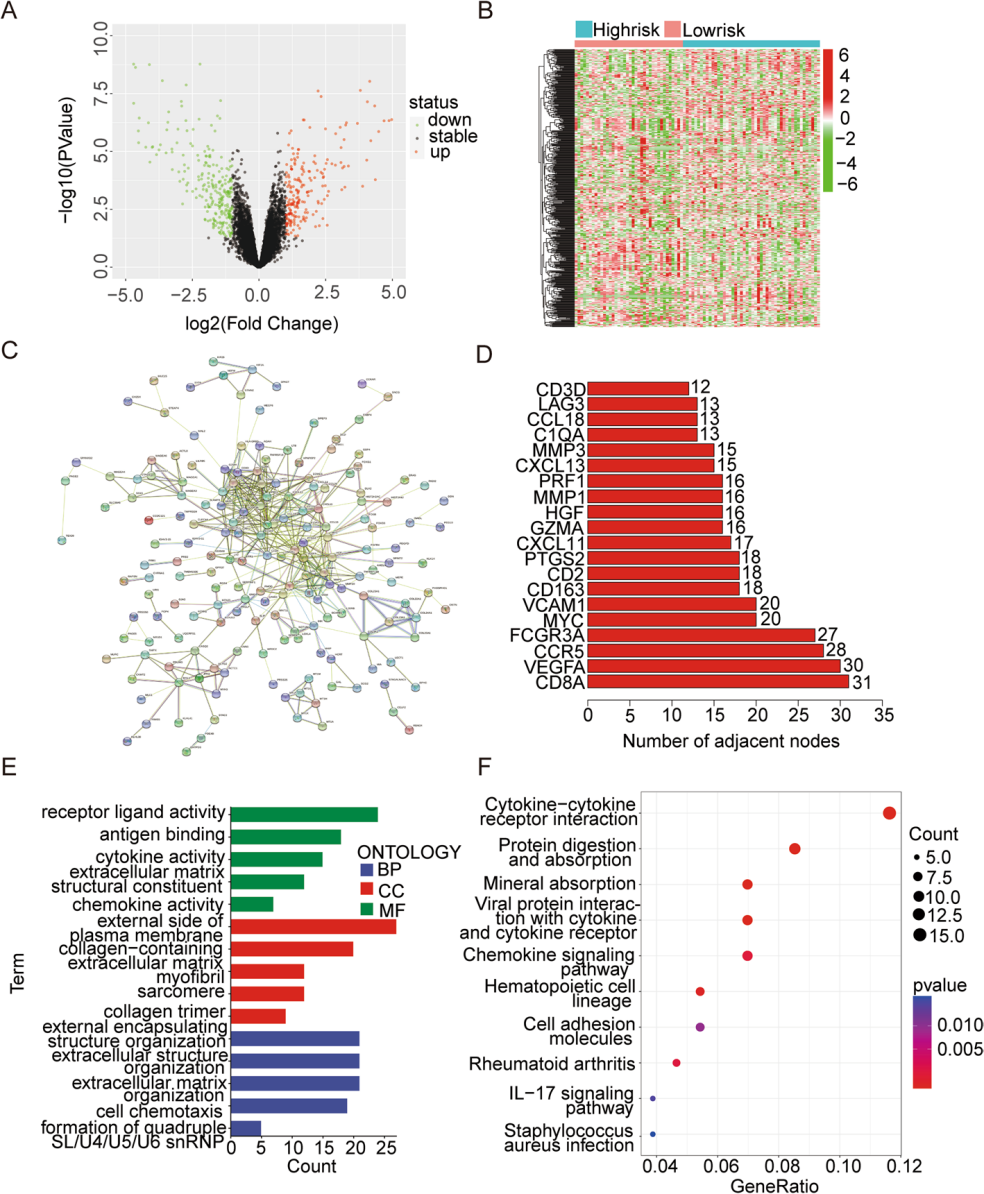
Gene differential analysis and functional enrichment analysis. **A** The volcano plot revealed differences in gene expression between high- and low-risk groups. **B** A heatmap was conducted using differently expressed genes in the two groups. **C** PPI network of those genes in the two groups. **D** The PPI network's hub genes. **E**, **F** GO and KEGG enrichment analysis

We analyzed the differentially expressed genes between the high- and low-risk groups in the entire TCGA cohort and created a volcano map (Fig. 7A). We found 472 differential genes between the two groups, of which 213 genes were upregulated and 259 genes were downregulated in the high-risk group. We created a heatmap of the expression of these differential genes in each sample and used it for subsequent analysis (Fig. 7B). We then used the STRING online database to analyze the expression patterns of the differential genes between the two risk groups (Fig. 7C). According to the PPI network diagram, *CD8A*, *VEGFA*, *CCR5*, *FCGR3A*, *MYC*, *VCAM1*, *CD163*, *CD2*, and *PTGS2* were considered hub genes (Fig. 7D), and their differentially expressed proteins had the greatest interaction correlation. Hub gene analysis showed that *CD8A*, *VEGFA*, *CCR5,* and *FCGR3A* were the top four genes in this PPI network. Then we performed functional analyses of these differential genes, including GO and KEGG analyses. We showed the GO enrichment analysis results in Fig. 7E. Biological process (BP) analysis mainly included the external encapsulating structure organization and cell chemotaxis. Cellular component (CC) analysis revealed the external side of the plasma membrane. Molecular function (MF) analysis mainly included antigen binding and receptor-ligand activity. Furthermore, KEGG enrichment analysis revealed some potential enrichment signaling pathways for these differentially expressed genes, including Viral protein interaction with cytokine and cytokine receptors, Protein digestion and absorption, Mineral absorption, and Hematopoietic cell lineage (Fig. 7F). The functional enrichment results of different genes in the two risk groups further confirmed that immune factors may play an indispensable role in osteosarcoma progression.

#### GSEA

**Fig. 8:**
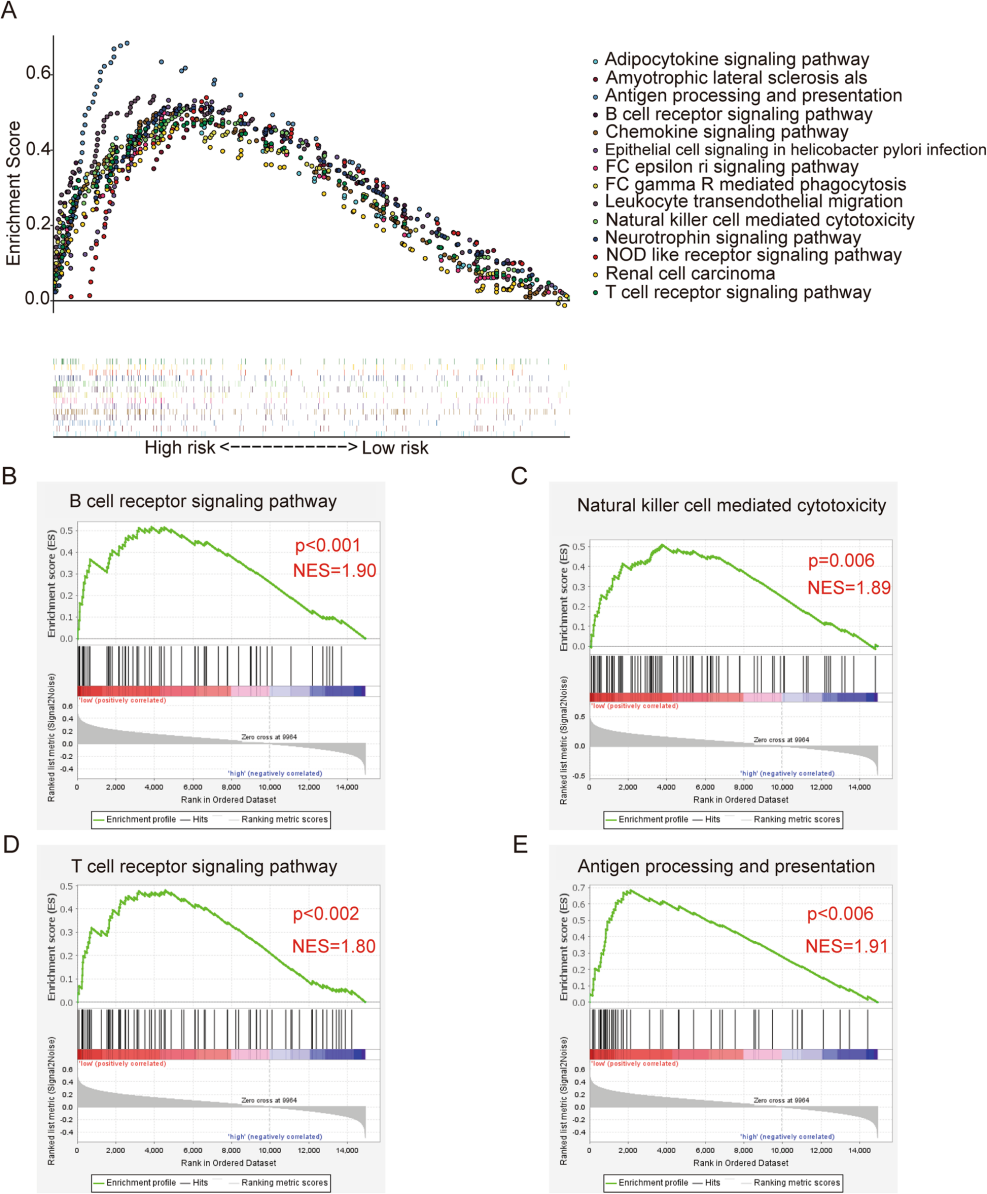
GSEA analysis of the two risk groups. **A** The top 14 changed pathways were conducted utilizing the KEGG gene set. **B-E** Select several immune-related pathways from the GSEA analysis. **B** B cell receptor signaling pathway. **C** Natural killer cell-mediated cytotoxicity. **D** T cell receptor signaling pathway. **E** Antigen processing and presentation

To more intuitively observe the information related to different signaling pathways between the two risk groups, we conducted a GSEA analysis and showed the top 14 functions and signaling pathways (Fig. 8A). These enrichment pathways revealed a variety of immune-related signaling pathways as compared to the high-risk group, including the B cell receptor signaling pathway (Fig. 8B), natural killer cell-mediated cytotoxicity (Fig. 8C), T cell receptor signaling pathway (Fig. 8D), and antigen processing and presentation, etc. (Fig. 8E), which were upregulated in the low-risk group. These enrichment results indicate that the high-risk group had a suppressed immune microenvironment, suggesting that immune factors play an important role in the development of osteosarcoma.

### Differences in immune cells and immune microenvironment between high—and low-risk groups in the TCGA entire cohort

**Fig. 9:**
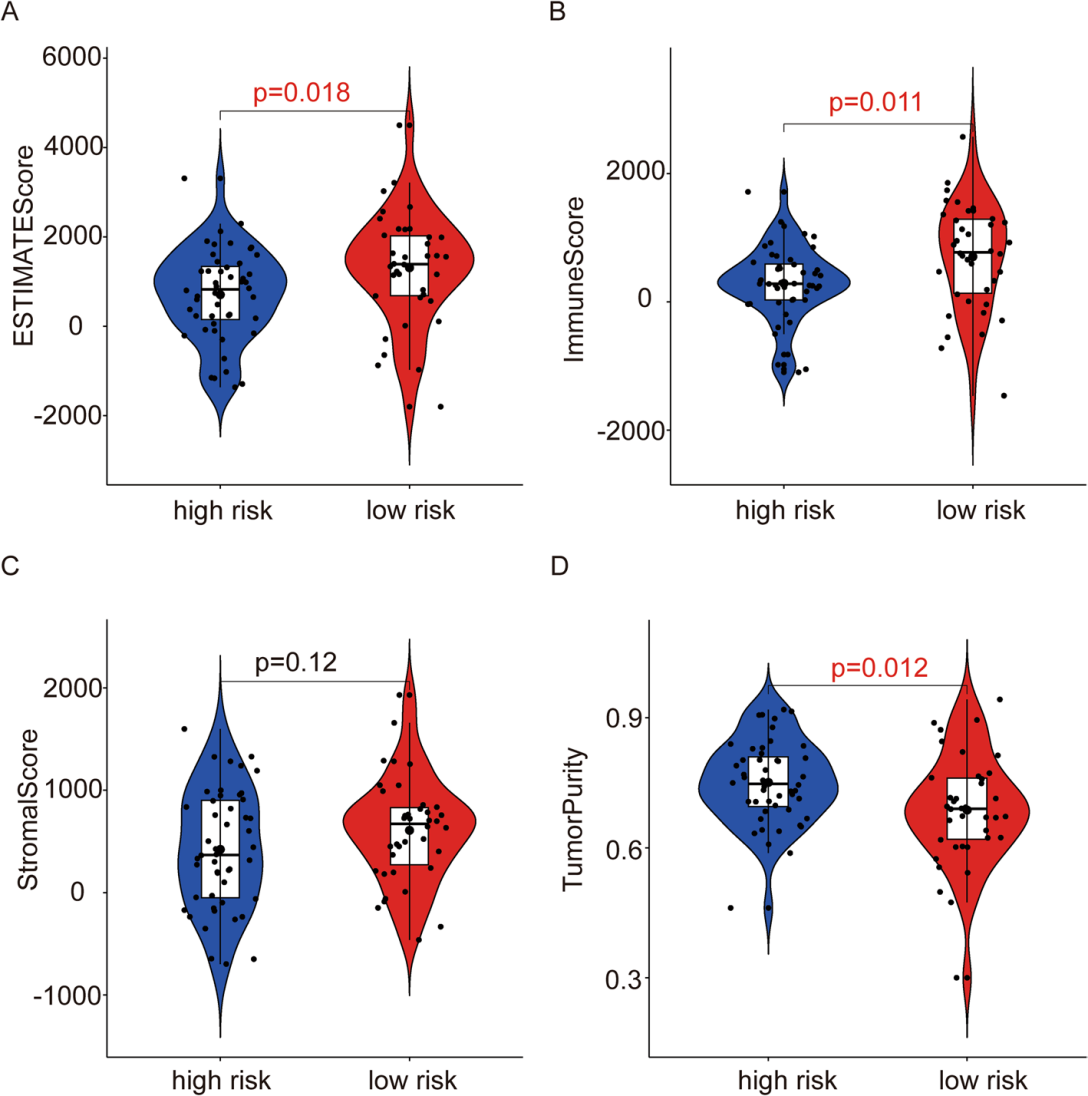
Analysis of immune-related scores for the two groups. **A-D** The stromal, immune, and estimate score, and tumor purity in high- and low-risk groups of the TCGA entire cohort. **A** The estimated score. **B** The Immune Score. **C** The Stromal Score. **D** The Tumor Purity

**Fig. 10:**
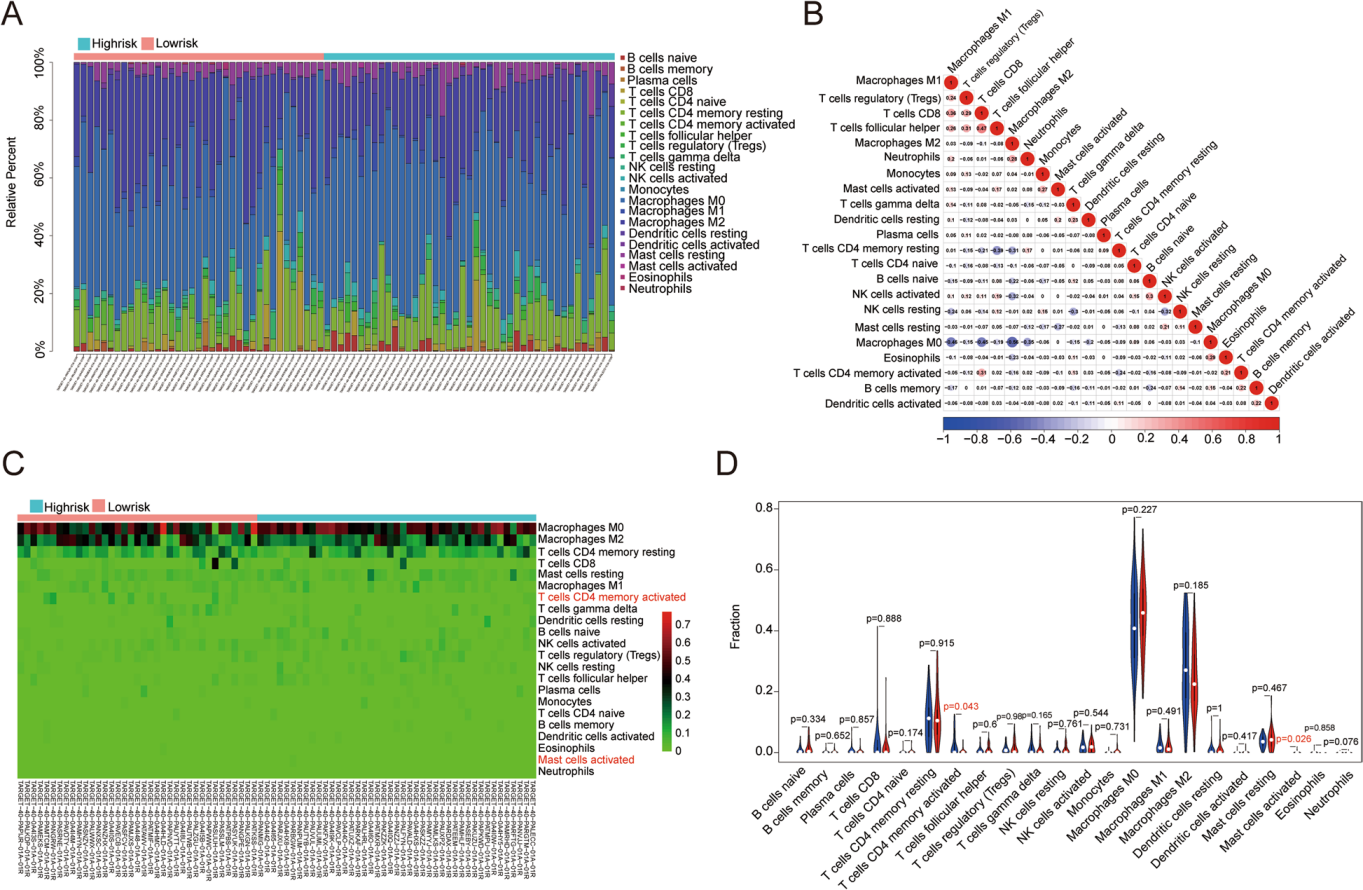
Comparison of immune cell infiltration in high- and low-risk groups in the TCGA cohort. **A** The relative quantity of immunocyte infiltration in the TCGA cohort. **B** The heatmap shows the correlation of immune infiltrating cells in the TCGA cohort. **C**, **D** The proportion of 22 immune cell types in high- and low-risk categories of the TCGA cohort

We used the estimation algorithm to calculate the immunological scores, estimate scores, stromal scores, and tumor purity of each sample in the TCGA entire cohort to better study the guiding effect of the prognostic model constructed using five genes in the *ARHGAP* family on the immune microenvironment. The results showed that patients in the high-risk group had significantly lower immunological scores, estimate scores, stromal scores, and higher tumor purity (Fig. 9A-D). The relationship between immune, estimate, stromal scores and tumor purity, and patient survival has previously been investigated [20]. Kaplan–Meier survival analysis revealed that patients with lower stromal, immune, and estimate scores had shorter overall survival, whereas patients with lower tumor purity had longer survival than those with higher tumor purity. This again proves that the immune status of the immune microenvironment of osteosarcoma can affect the occurrence and development of osteosarcoma, as well as the survival of patients with osteosarcoma. This suggests that the five genes of the *ARHGAP* family used to construct prognostic models affect the immune microenvironment of patients and play a role in the immunotherapy of osteosarcoma. To investigate the relationship between *ARHGAP* gene expression and immune infiltration in osteosarcoma, we used CIBERSORT to calculate the proportion of immune cell infiltration in each sample in the TCGA dataset and compared it between the two risk groups. We then plotted a bar graph (Fig. 10A). The bar graph shows that Macrophages *M2*, Macrophages *M0,* and *CD4* memory resting T cells are the most abundant of these immune cells in the immune microenvironment of all osteosarcoma samples. Moreover, the increase in Macrophages *M2* is associated with the occurrence and development of tumors and poor prognosis of patients, and our results confirm this view. We also investigated the relationship between individual immune cells in the immune microenvironment (Fig. 10B). The results showed a relatively large correlation between macrophages and T cells, suggesting that there might be a mechanism of mutual regulation between the two types of cells in the immune microenvironment, which could provide ideas for subsequent analysis of the immune microenvironment and immunotherapy of osteosarcoma. We also performed an immune infiltration analysis and analyzed the differences in the expression of immune cells between the risk groups (Fig. 10C-D). According to the results of the study, the proportion of immune cells in the immunological microenvironment of the two risk groups of osteosarcoma patients was different.

### ssGSEA

**Fig. 11:**
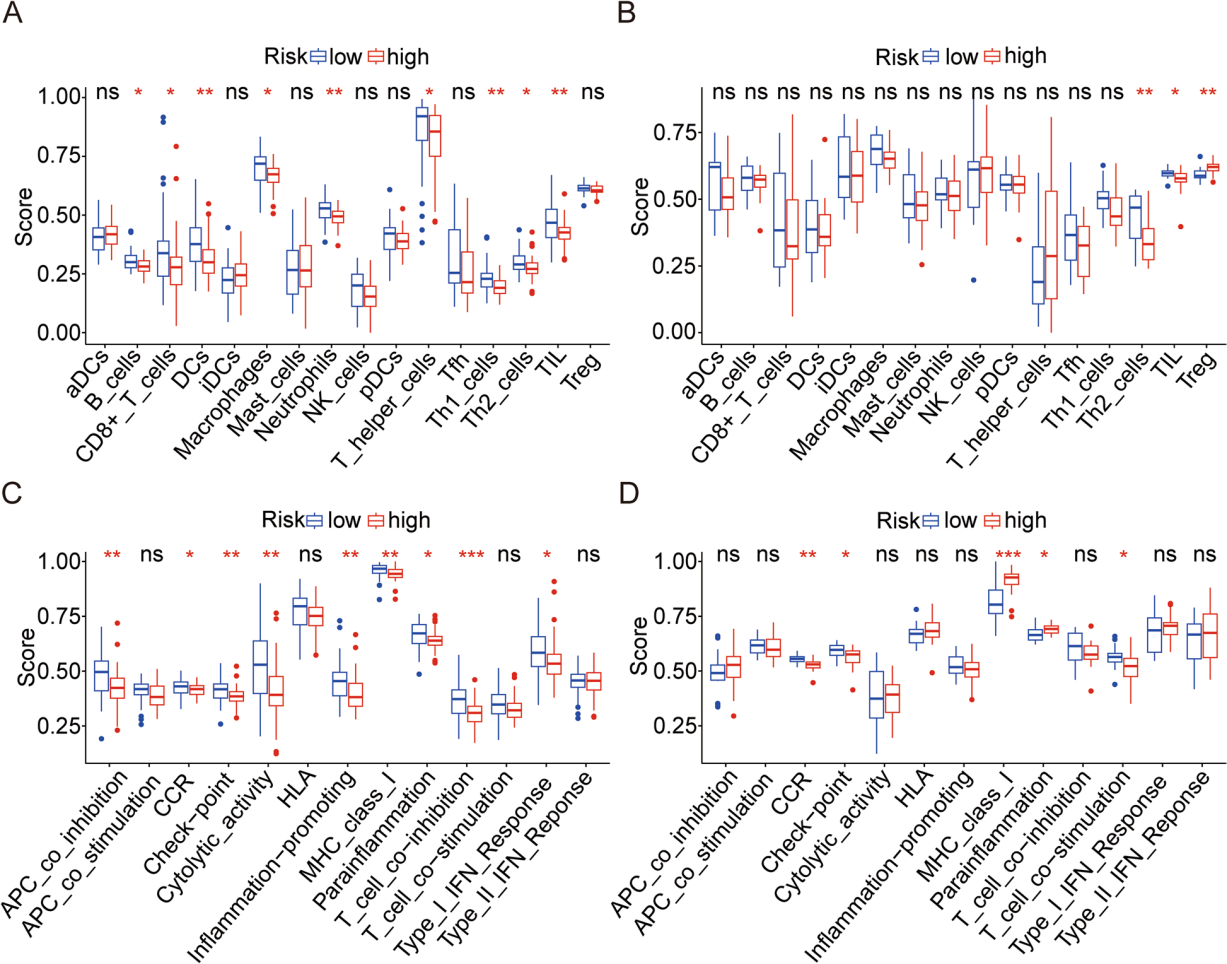
Comparison of immune cell infiltration and immune function based on ssGSEA. **A**, **B** Box plots exhibiting enrichment scores of immunocytes between the two subgroups in TCGA and GSE39055 cohorts. **C**,** D** Box plots exhibiting enrichment scores of the related immune function between the two subgroups in TCGA and GSE39055 cohorts

Based on ssGSEA, we analyzed differences in immune function scores and immune cell enrichment scores between the two groups. The low-risk group had much more *Th2* cells and TIL cells than the high-risk group, which was consistent with the findings from the TCGA and GSE39055 cohorts (Fig. 11A-B). In addition, in the TCGA and GSE39055 cohorts, the low-risk group's immune cell concentration was much higher than that of the high-risk group, especially *DCs*, *Th1* cells, and Neutrophils, suggesting that the deficiency of immune function is an important factor leading to poor prognosis in patients with osteosarcoma. In the TCGA and GSE39055 cohorts, higher *CCR* and checkpoint scores in the low-risk group than those in the high-risk group (Fig. 11C-D) mainly manifested the differences in immune function between the two risk groups.

### Kaplan–Meier survival analysis of the five ARHGAP genes in the two risk groups

**Fig. 12:**
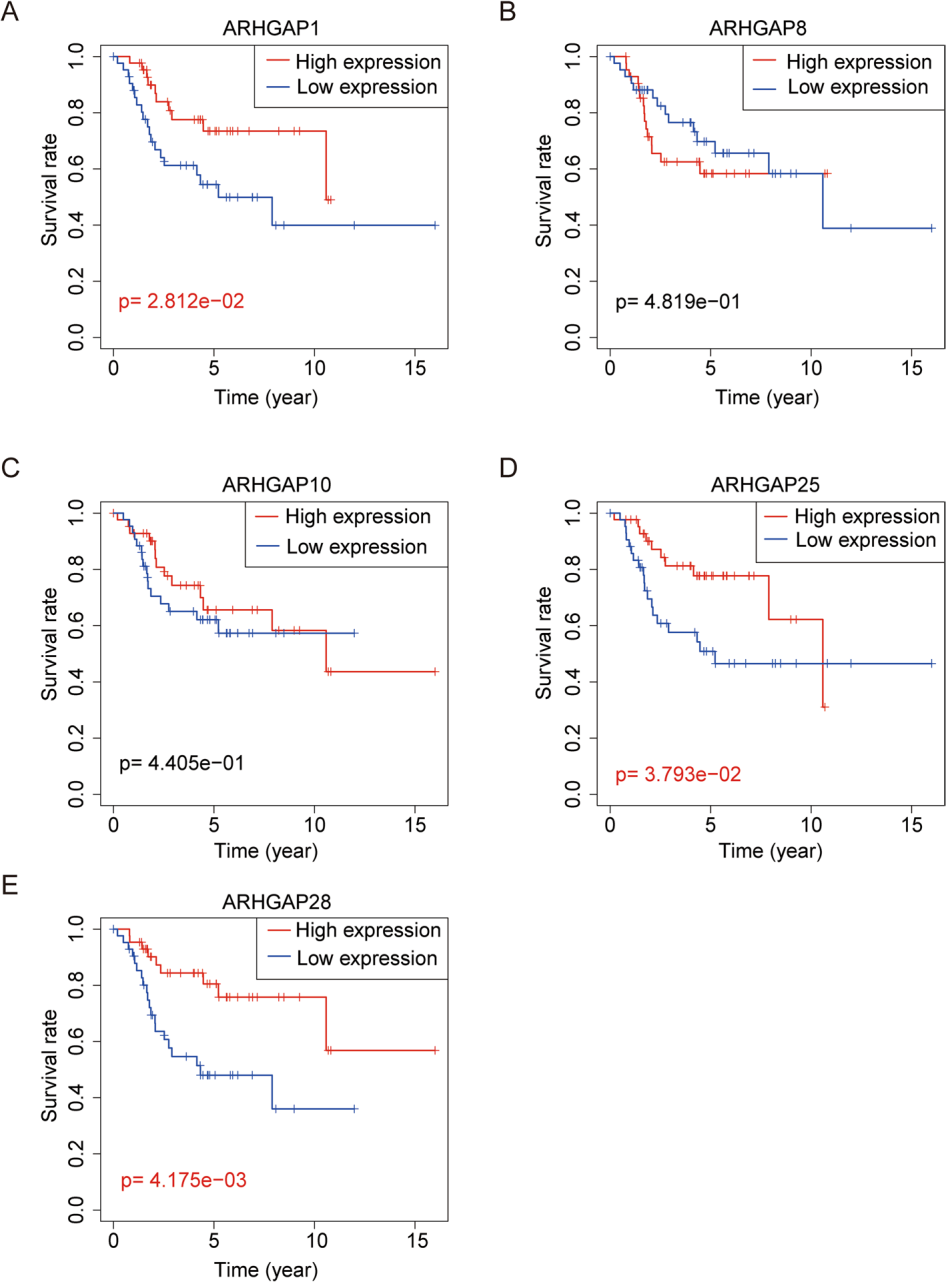
Kaplan–Meier survival analysis of the five *ARHGAP* genes in high- and low-risk groups. **A** Kaplan–Meier survival analysis of *ARHGAP1*. **B** Kaplan–Meier survival analysis of *ARHGAP8*. **C** Kaplan–Meier survival analysis of *ARHGAP10*. **D** Kaplan–Meier survival analysis of *ARHGAP25*. **E** Kaplan–Meier survival analysis of *ARHGAP28*

Finally, we performed a Kaplan–Meier curve survival analysis of the five genes that constructed the prognosis model in the TCGA cohort. Kaplan–Meier curves showed that high expression of *ARHGAP1*, *ARHGAP25*, and *ARHGAP28* was associated with better prognosis, whereas expression of *ARHGAP8* and *ARHGAP10* was not associated with patient prognosis (*p* < 0.05) (Fig. 12A-E).

### Overexpression of ARHGAP28 inhibits the proliferation, migration, and invasion of human osteosarcoma cell lines

**Fig. 13:**
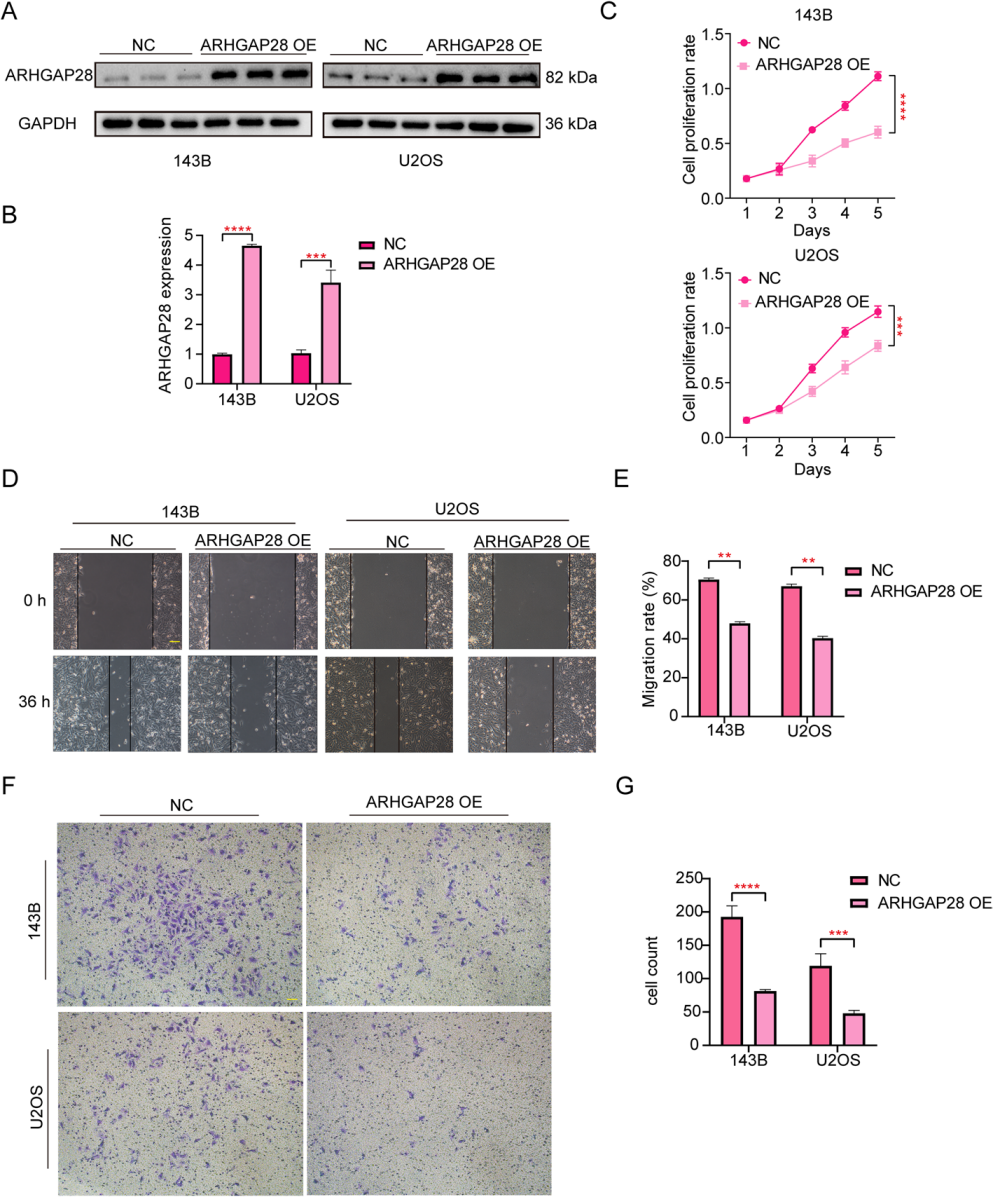
Overexpress *ARHGAP28* inhibits the proliferation, migration, and invasion of human OS cell lines. **A**, **B** The protein content of *ARHGAP28*. **C** CCK-8 assay was applied to measure the viability of osteosarcoma cells in the *ARHGAP28* overexpression group, and NC group. **D** Photos of 0 h and 36 h wound healing assay. **E** Statistical analysis of migration rate. **F-G** The transwell assay was applied to measure the invasion ability of osteosarcoma cells in the two groups. Scale bar: 400 µm. All data are from three independent experiments and are shown as mean ± SD. “**” represented “*p* < 0.01”, “***” represented “*p* < 0.001”, “****” represented “*p* < 0.0001”

To verify the bioinformatics results we made earlier, we conducted a western blot experiment, CCK-8 experiment, transwell experiment, and wound healing assay. We found that overexpression of *ARHGAP28* inhibited the activity of human osteosarcoma cells. According to the results of western blot, we successfully overexpressed *ARHGAP28*, the protein content of *ARHGAP28* was about 150% higher than that of the NC groups (Fig. 13A-B), and then we used the overexpressed cell lines as well as the NC group for follow-up experiments. Compared with the NC group, the cell viability of the *ARHGAP28* overexpression group was significantly decreased (Fig. 13C). Results of wound healing experiments showed that *ARHGAP28* inhibited the migration of human osteosarcoma cell lines (Fig. 13D-E). In addition, transwell results suggested that *ARHGAP28* overexpression could also inhibit the invasion ability of osteosarcoma cell lines (Fig. 13F-G). The above results verified the correctness and accuracy of our previous analysis. High expression of *ARHGAP28* in osteosarcoma cell lines can inhibit the proliferation, migration, and invasion of osteosarcoma, and we can judge the risk of OS patients based on this.

### *Increased expression of ARHGAP28 inhibits tumor growth *in vivo

**Fig. 14:**
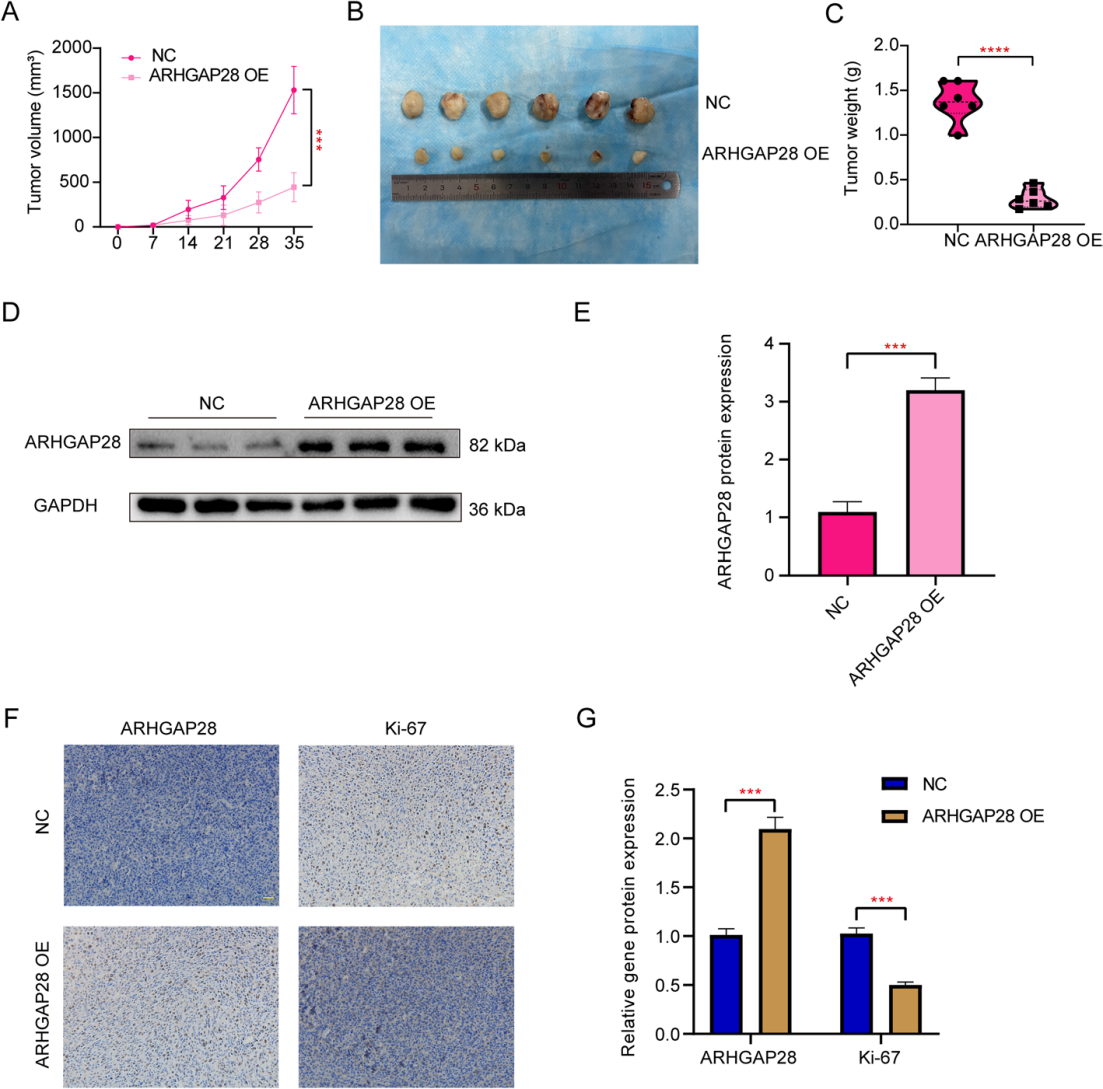
Increased expression of *ARHGAP28* inhibits tumor growth in vivo. **A** Tumor growth was measured in vivo by monitoring its volume every 7 days.** B** The xenograft tumor size in nude mice was compared. **C** The weight of tumors was compared between the two groups. **D-E** The xenograft tumor was subjected to western blot and quantitative analyses of *ARHGAP28*. **F**, **G** Immunohistochemical analysis of *ARHGAP28* and Ki-67 in tumors excised from two groups. Scale bar: 200 µm. All data are from three independent experiments and are shown as mean ± SD. “***” represented “*p* < 0.001”, “****” represented “*p* < 0.0001”

We injected stable *ARHGAP28* overexpressing 143B osteosarcoma cells and NC cells into nude mice subcutaneously (Fig. 14D-E). Tumor volume was monitored every 7 days, and *ARHGAP28* overexpression resulted in smaller tumors compared to NC (Fig. 14A-C). Moreover, IHC results suggested a lower Ki-67 level in the *ARHGAP28* overexpression group than in the NC group (Fig. 14F-G). In conclusion, our results indicate that *ARHGAP28* overexpression suppresses in vivo tumor growth.

## Discussion

Osteosarcoma is the most common primary malignant bone tumor in orthopedics, with two peaks in adolescents and the elderly. The peak age of adolescent-onset is about 15 years old, mainly primary OS, and the second peak age is about 75 years old, mainly secondary OS [21]. OS mainly occurs in the epiphyseal region of the long diaphysis, where blood transport is abundant [1]. In the early stage of osteosarcoma, blood transport to the lung is most common and develops rapidly, which greatly reduces the survival of patients with osteosarcoma [22]. With the increase and improvement of treatment methods, the survival rate of patients with osteosarcoma has been greatly improved, but how to further improve the prognosis of patients is a major clinical challenge, especially for patients with pulmonary metastatic osteosarcoma, which occurs earlier and has a worse prognosis [23]. Hence, on the one hand, we should be looking for more effective treatments; on the other hand, we should also develop some new ideas. We can use high-throughput sequencing technology and existing sequencing results to screen genes, and then predict the prognosis of patients so that we can carry out more personalized treatment for patients.

For nearly 20 years, we have considered the *Rho* family as an anti-tumor target, especially for *RAS*-driven tumors. *Rho* family proteins transition between active GTP-binding states and inactive GDP-binding states, which are regulated by the *ARHGAP* family and can increase the intrinsic GTPase activity of *Rho* GTPase to convert it to inactive GDP-binding states [11]. Nowadays, it is believed that most *ARHGAP* genes have multiple functional domains except the *RHOGAP* functional domain, which integrates signal factors in many signaling pathways and may mediate the interaction between the *Rho* family and other signaling pathways. However, the number of *ARHGAP* proteins is much larger than that of their substrate *Rho* proteins, and *ARHGAP* proteins have diverse biological functions. Therefore, the deep regulatory mechanism of *ARHGAP* on the *Rho* family is still far from clear. The *ARHGAP* family is involved in many biological activities, such as exocytosis, endocytosis, cytokinesis, cell differentiation, cell migration, neuronal morphogenesis, angiogenesis, and tumor suppression [11]. In recent years, the relationship between *ARHGAP* family genes and tumor development, invasion, and metastasis has attracted more and more attention [24]. In this study, we screened the *ARHGAP* family genes and finally screened 5 genes to construct a risk model for evaluating the prognosis of patients with osteosarcoma, and to provide certain ideas and help for clinical treatment.

In this study, we first collected gene expression information and clinical information of patients with osteosarcoma in the TARGET cohort and the GSE39055 cohort and then removed patients without follow-up information and survival status to prepare the data for our next analysis. For better analysis, we then obtained the genetic information of the *ARHGAP* family from the online website, and uniformly named it in the two datasets. We then performed univariate COX analysis in the TCGA cohort and screened out 9 genes associated with the prognosis of patients with osteosarcoma. For subsequent analysis and verification, we randomly divided patients in the TCGA cohort into two groups at a ratio of 1: 1, namely the training cohort and the testing cohort. We performed LASSO regression analysis and multivariate COX regression analysis on the patients in the training cohort and finally obtained five genes: *ARHGAP1*, *ARHGAP8*, *ARHGAP10*, *ARHGAP25,* and *ARHGAP28*, and then we constructed a risk prediction model for patients with osteosarcoma based on these five genes. We calculated each patient's risk score and divided them into high- and low-risk groups, and then analyzed the relationship between survival status and survival time and risk score in the training cohort, testing cohort, entire internal cohort, and GSE39055 cohort respectively, finally, Kaplan–Meier survival analysis and ROC curve were performed for verification. The results show that the high-risk group had a lower survival time than the low-risk group. This suggests that our risk model can well predict the prognosis of patients with osteosarcoma. We can tell that these genes are protective factors for patients with osteosarcoma. Among them, *ARHGAP1*, *ARHGAP8,* and *ARHGAP10* play different roles in different tumors or different pathways. *ARHGAP1* was the first gene discovered in this family, and its content in cervical cancer cells and Ewing Sarcoma (ES) cells was lower than that in the matching normal tissue, which proved that it could inhibit the cell vitality, cell migration, and invasion of these two cancer cells in a time-dependent manner to a certain extent [25, 26]. However, in breast cancer (BC), *ARHGAP1* is a carcinogenic factor, and its expression level in BC samples is higher than that in normal tissues, its overexpression can promote the proliferation and invasion of BC cells while inhibiting its expression can significantly inhibit the growth of tumors [27–29]. *ARHGAP1* may also regulate the bone microenvironment by inhibiting the *RhoA/ROCK* pathway, which stimulates osteogenic differentiation of mesenchymal stem cells [30]. However, the role of *ARHGAP1* in osteosarcoma has not been reported, which can be further studied in the future. Similarly, *ARHGAP8* is overexpressed in most colorectal cancers compared to normal tissues, but we observe relatively low expression in Bladder cancer, suggesting that *ARHGAP8* may play different roles in different tumors, but its role in osteosarcoma is unknown [31, 32]. *ARHGAP10* is well known as a tumor suppressor and has been demonstrated in a variety of cancers, such as Uterine leiomyomas (ULs), prostate cancer, ovarian cancer (OC), lung cancer, colon carcinoma (CRC) and BC [33–38]. *Cdc42*, a key protein that cancer cells need to metastasize, helps them spread through the bloodstream to other parts of the body. In ovarian cancer, *RHGAP10* inhibits *Cdc42* activity in cells, in turn, it can inhibit the growth and invasion of tumors, thus playing a role in cancer suppression [37]. However, *ARHGAP10* manifested as an oncogene in gastric tumors and non-small cell lung cancer (NSCLC) [39–41]. The expression level of *ARHGAP10* in NSCLC is higher than that in normal tissues. When its expression is decreased, the expression of *GLUT1* is also decreased, which inhibits the glucose metabolism process of cells and thus the progression of cancer [40]. Therefore, the role of *ARHGAP10* in different tumors may be related to its participation in different pathways or different regulatory molecules upstream and downstream of the same pathway. However, its specific role in osteosarcoma is still unclear, so we can conduct further research. *ARHGAP25* has also been widely studied as a tumor suppressor gene in cancer, including Pancreatic adenocarcinoma (PAAD), NSCLC, Lung cancer, and CRC [14, 42–46]. Epithelial-mesenchymal transition (EMT) is a common mechanism of tumor metastasis, which can reduce the adhesion between cells so that tumor cells can be separated from the original site to metastasize [47]. The *Wnt/β-catenin* pathway can increase the viability and invasion ability of cancer cells by activating EMT [48]. However, *ARHGAP25* exerts its anticancer effects by negatively regulating EMT and *Wnt/β-catenin* pathways [44]. Of course, *ARHGAP25* may regulate different pathways in different tumors to play a role in cancer inhibition. However, whether *ARHGAP25* can inhibit the metastasis of osteosarcoma has not been studied, and it can become the object of our subsequent research [49]. Finally, the expression level of *ARHGAP28* in osteosarcoma is significantly related to the prognosis and survival time of patients, but it has not been studied in osteosarcoma. Therefore, we speculate that *ARHGAP28* is a tumor suppressor gene for osteosarcoma, and we plan to further study it in the next step.

We then constructed a nomogram based on the TCGA cohort to incorporate age, gender, and risk scores and simulated 2-, 3-, and 5-year time-dependent AUC curves for osteosarcoma patients in the TCGA and GSE39055 cohorts, respectively. The results showed that the risk score was an independent predictor of the prognosis of patients with osteosarcoma, and the simulation results of the AUC curve were good, which could better prove the accuracy and applicability of the model. To find out which functional pathway the molecular differences between the high- and low-risk groups are enriched in, to better screen the differential genes that can be used as targets for clinical diagnosis and treatment, we screened the differential genes between the high- and low-risk groups and conducted functional enrichment analysis. KEGG results show that it mainly enriched them in Protein digestion and absorption and Cytokine-cytokine receptor interaction, which also confirms our above analysis. They interact with different upstream and downstream molecules in different tumors to show different functions, and the process is very complex [11].

Nowadays, immunotherapy has been emphasized in the treatment of patients with osteosarcoma [50]. Therefore, we further studied whether they relate the risk model to the immune microenvironment, which can provide some ideas for the immunotherapy of osteosarcoma. GSEA analysis showed that the low-risk group had higher enrichment of immune function than the high-risk group [51], such as the B cell receptor signaling pathway, natural killer cell-mediated cytotoxicity, T cell receptor signaling pathway, and antigen processing and presentation. They have reported that Macrophages *M2* can promote the generation of tumors [52–54]. In our study, the content of Macrophages *M2* is relatively high in osteosarcoma. ssGSEA showed that the content of immune cells in the high-risk group was much lower than that in the low-risk group, indicating that the occurrence and development of osteosarcoma are closely related to the immune environment, which will provide new ideas for us to find new therapeutic targets and methods for osteosarcoma in the future.

Since the role of *ARHGAP28* in osteosarcoma remains unclear, we confirmed the role of *ARHGAP28* through in vitro and in vivo biological experiments. Overexpression of *ARHGAP28* had significant effects on the viability, proliferation, migration, and invasion of OS cells. We found that overexpression of *ARHGAP28* can inhibit the proliferation, migration, and invasion of osteosarcoma cells. In vivo experiments have shown that overexpression of *ARHGAP28* can inhibit tumor growth in mice, and IHC has shown that the reduced level of Ki-67 in the *ARHGAP28* overexpression group can inhibit the proliferation of tumor cells. In summary, *ARHGAP28* may play a positive role in inhibiting the growth and progression of osteosarcoma.

However, inevitably, our research also has some shortcomings. First, we only used an external GSE39055 cohort for verification, which may have some discrepancies in some data sets. Second, the expression levels of *ARHGAP1*, *ARHGAP8*, and *ARHGAP10* in our model showed the same trend with the prognosis and survival time of patients with osteosarcoma, but there was no significant correlation. Whether the model constructed by combining these five genes is also applicable to other cohorts needs further verification. Third, we lack clinical samples to verify the accuracy of the model we constructed, so we can only test our hypothesis with cell experiments. Finally, we did not investigate *ARHGAP28 further*, such as its relationship to human immunity.

The study categorized OS invalids into risk groups based on the *ARHGAP* family. The high-OS group displayed abnormal immune function, such as the B cell receptor signaling pathway, natural killer cell-mediated cytotoxicity, T cell receptor signaling pathway, and antigen processing and presentation. The results show that *ARHGAP* family genes are likely to play a role in the immune function of the human body, inhibiting the occurrence and progression of tumors, and these gene targets may also be promising personalized drug targets.

In summary, we constructed a five-gene (*ARHGAP1*, *ARHGAP8*, *ARHGAP10*, *ARHGAP25,* and *ARHGAP28*) risk prognostic model based on the *ARHGAP* family. It can predict the prognosis of patients with osteosarcoma, and verify its accuracy and universality. Finally, we also analyzed the relationship between it and the immune system of patients, which provided ideas and directions for our follow-up research and the management and treatment of clinical patients.

### Supplementary Information

**Additional file 1.**

**Additional file 2.**

## Data Availability

We obtained the datasets analyzed and generated during the current study in the TCGA GDC repository (https://portal.gdc.cancer.gov) and GEO repository (https://www.ncbi.nlm.nih.gov/geo/).
