# Supplementary material for: Construction of a prognostic risk score model based on the ARHGAP family to predict the survival of osteosarcoma
Source: BMC Cancer. 2023 Dec 1;23:1179. doi: 10.1186/s12885-023-11673-w (PMC10693137; doi:10.1186/s12885-023-11673-w)
Supplement: Supplementary file 1 — Additional file 1. [file 12885_2023_11673_MOESM1_ESM.docx]

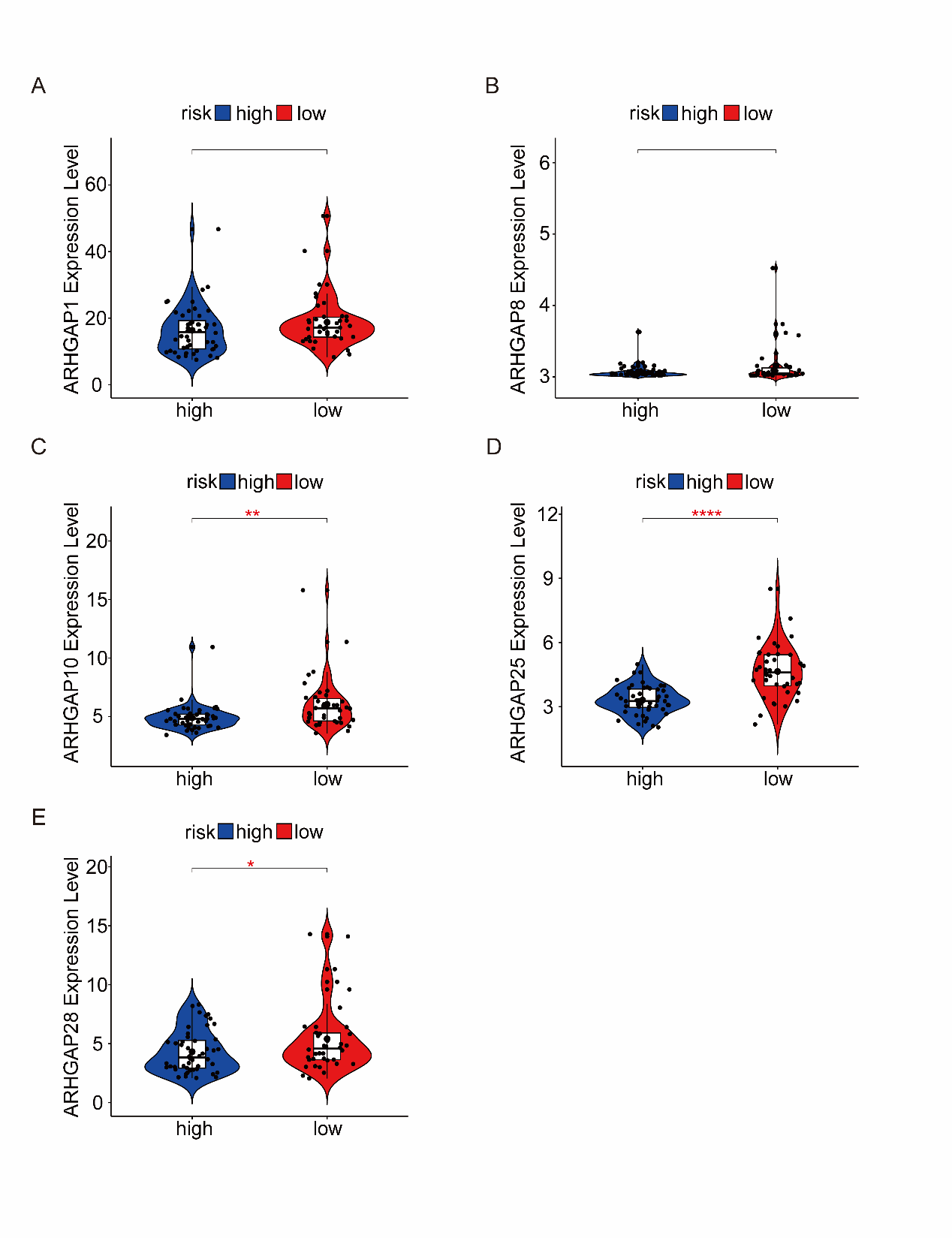


**Additional file 1: Fig. S1 |** Expression analysis of the five *ARHGAP* genes in high- and low-risk groups. **(A)** The expression of *ARHGAP1*. **(B)** The expression of *ARHGAP8*. **(C)** The expression of *ARHGAP10*. **(D)** The expression of *ARHGAP25*. **(E)** The expression of *ARHGAP28*. “*” represented “p<0.05”, “**” represented “p<0.01”, “****” represented “p<0.0001”.
